# Supplementary material for: Exploring Transcriptomic Landscapes in Red Blood Cells, in Their Extracellular Vesicles and on a Single-Cell Level
Source: Int J Mol Sci. 2022 Oct 25;23(21):12897. doi: 10.3390/ijms232112897 (PMC9657499; doi:10.3390/ijms232112897)
Supplement: Supplementary file 1 [file ijms-23-12897-s001.zip › Supplementary Methods and Figures S1-S5.pdf]

Supplementary Material

Kerkelä E, Lahtela J, Larjo A, Impola U, Mäenpää L, Mattila P: Exploring transcriptomic landscapes in red blood cells, in their extracellular vesicles and on a single-cell level

Supplementary Methods

Characterization of RBC-EVs with Western Blot: Pelleted RBC-EV samples (8d, n=4) were characterized by Western Blot for the origin of EVs. Equal amount of protein (9 µg) was loaded and run on TGX Mini-Protean 4-20% gel (Bio-Rad Laboratories, Hercules, CA, USA) and subsequently electrophoretically transferred to nitrocellulose membranes (BioRad). Nonspecific binding was blocked with 6 % milk-0.05 % Tween 20-Tris (room temperature, 1 h). Rabbit anti-human CD235a (1:1000 dilution, clone EPR8200, Abcam, Cambridge, UK), mouse anti-human Hb (1:10000, clone 7E1F, Abcam), mouse anti-human CD61 (2µg/ml, clone VI-PL2, BD Biosciences), and mouse anti-human apolipoprotein A1 (Apo-A1; 1:1000, Medix Biochemica, Espoo, Finland) antibodies were incubated in 2 % milk-0.05% Tween 20-Tris (+4 °C, overnight), followed by incubation with a horseradish peroxidase-conjugated goat anti-rabbit and goat anti-mouse IgG (H+L) secondary antibodies (1:3000 dilution, 1 h, RT, Bio-Rad Laboratories). The enhanced chemiluminescence (ECL) reagent/kit (GE Healthcare, Amersham, Little Chalfont, UK) was used for detection of results. Apo-A1 and CD61 expression was also studied from supernatant of a 2-day old platelet concentrate (SN) and platelet EV aliquot (PLT-EV), collected by ultracentrifugation from 5d-old platelet concentrate, respectively, to confirm the functionality of antibodies.

Scanning electron microscopy: EVs were collected from RBC concentrate, aged 23d of storage and processed the same day. In total, 50 µL of concentrated EVs was placed on concanavalin A (Sigma-Aldrich)–coated glass cover slips and fixed with 5% glutaraldehyde (G7651 Sigma, EM quality) in 0.05 M HEPES buffer (pH 7.2) for 30 min and washed repeatedly with 0.05 M HEPES buffer and stored in a buffer solution until the next day. Samples were post-fixed with 1% OsO_4 and dehydrated in an increasing gradient of ethanol. Samples were allowed to dry on hexamethyldisilazane and were imaged immediately. Before microscopy, the samples were coated with platinum. SEM analyses were conducted by means of a FEG-SEM Quanta 250 microscope (FEI Company, Hillsboro, OR, USA).

Supplementary Figure Legends

Supplementary Figure S1. (**A**) Gene body coverage plots from the transcription start site (5') to the transcription end site (3') for RBC and EV samples were generated with the RSeQC toolkit. (**B**) Principal component analysis (PCA) of RBC (blue) and EV (red) samples. The percentages on the axes denote the ratio of explained variance.

Supplementary Figure S2. Correlation plots calculated in logarithmic (log2) scale of TPM (Transcripts Per Kilobase Million) values for 3 representative RBC/RBC pairs, 3 representative EV/EV pairs and all relevant RBC/EV pairs. Correlation coefficient R^2 is shown for each plot.

Supplementary Figure S3. RBC-EV characterization. (**A**) Western blot analysis of representative d8 RBC-EV samples shows that CD235a and Hb proteins are clearly detectable in RBC-EVs, while CD61 and ApoA1 are not present with the same exposure time. Original membrane was cut in four pieces based on predicted size of the protein and incubated individually with the corresponding antibodies (red lines depict the borders of the pieces). (**B**) Apo-A1 was detected from supernatant of a 2-day old platelet concentrate (SN) and CD61 was expressed in platelet EVs (PLT-EV) collected from 5d-old platelet concentrate, which confirmed that antibodies negative in RBC-EVs were functional. (**C**) Image of an stain free gel shows, that equal amount of protein (9 µg) was loaded per lane. (**D**) Typical size distribution pattern of RBC-EVs show that the majority of EVs are 100-300 nm in diameter as measured by nanoparticle tracking analysis (NTA). (**E**) Scanning electron microscopy reveals the wide variety of shapes and sizes in EVs from RBC concentrate.

Supplementary Figure S4. Violin plots showing the distribution of the total number of genes (nGene), the total UMIs (nUMI) and the percentage of mitochondrial gene expression (percent.mito) per cell for the total population of cells prior bioinformatics exclusion.

Supplementary Figure S5. Combined list of expressed genes (5 or more UMIs) from single-cell sequencing analysis (158 in total, gene list in Supplementary Table 8) was compared with "bulk" RBC transcriptome using the Venny 2.1.0 tool and Ensembl gene IDs.

Figure S1.

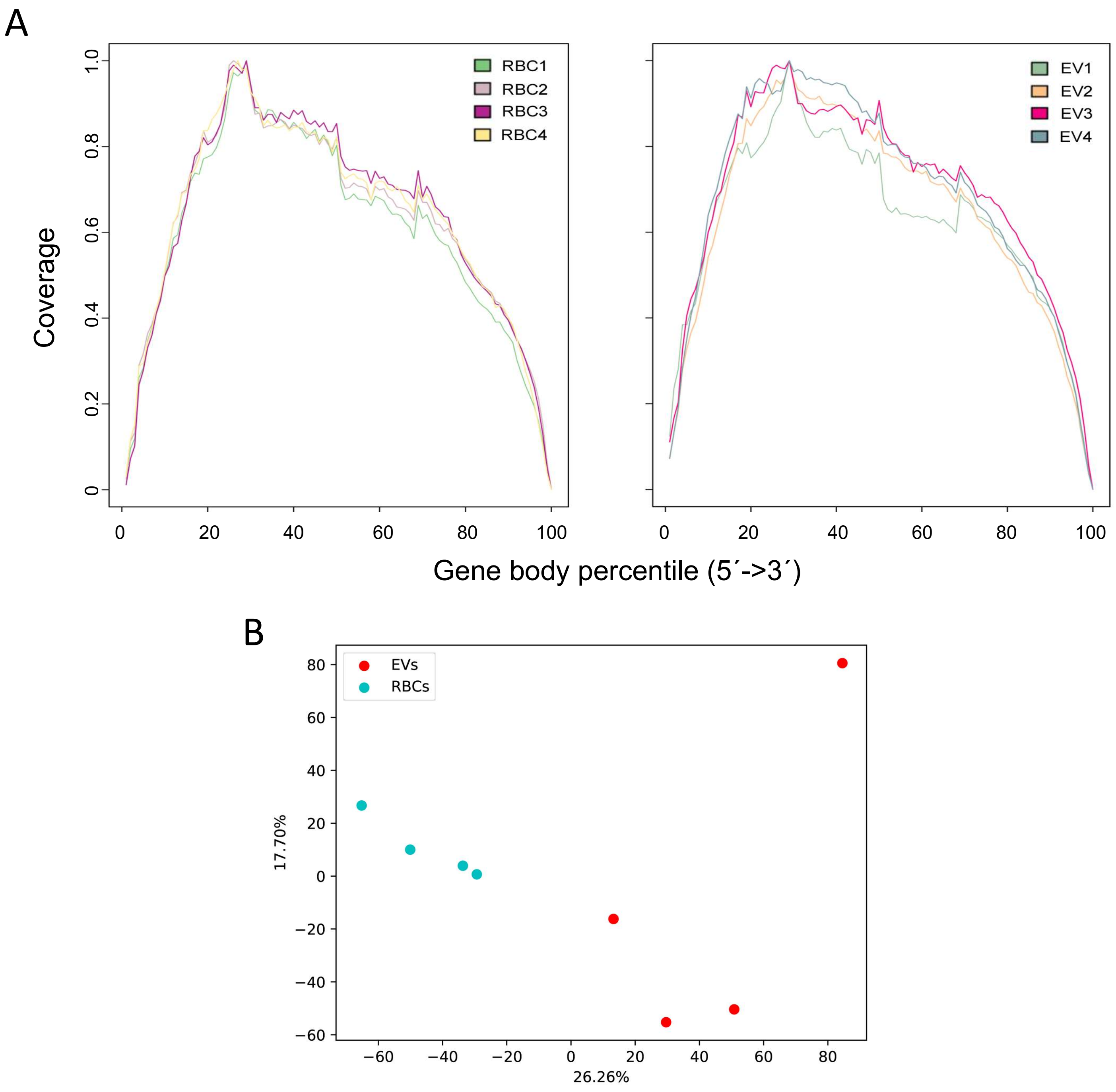
A
RBC1
RBC2
RBC3
RBC4
EV1
EV2
EV3
EV4
Coverage
Gene body percentile (5´->3´)
B
EVs
RBCs
17.70%
26.26%

Figure S2.

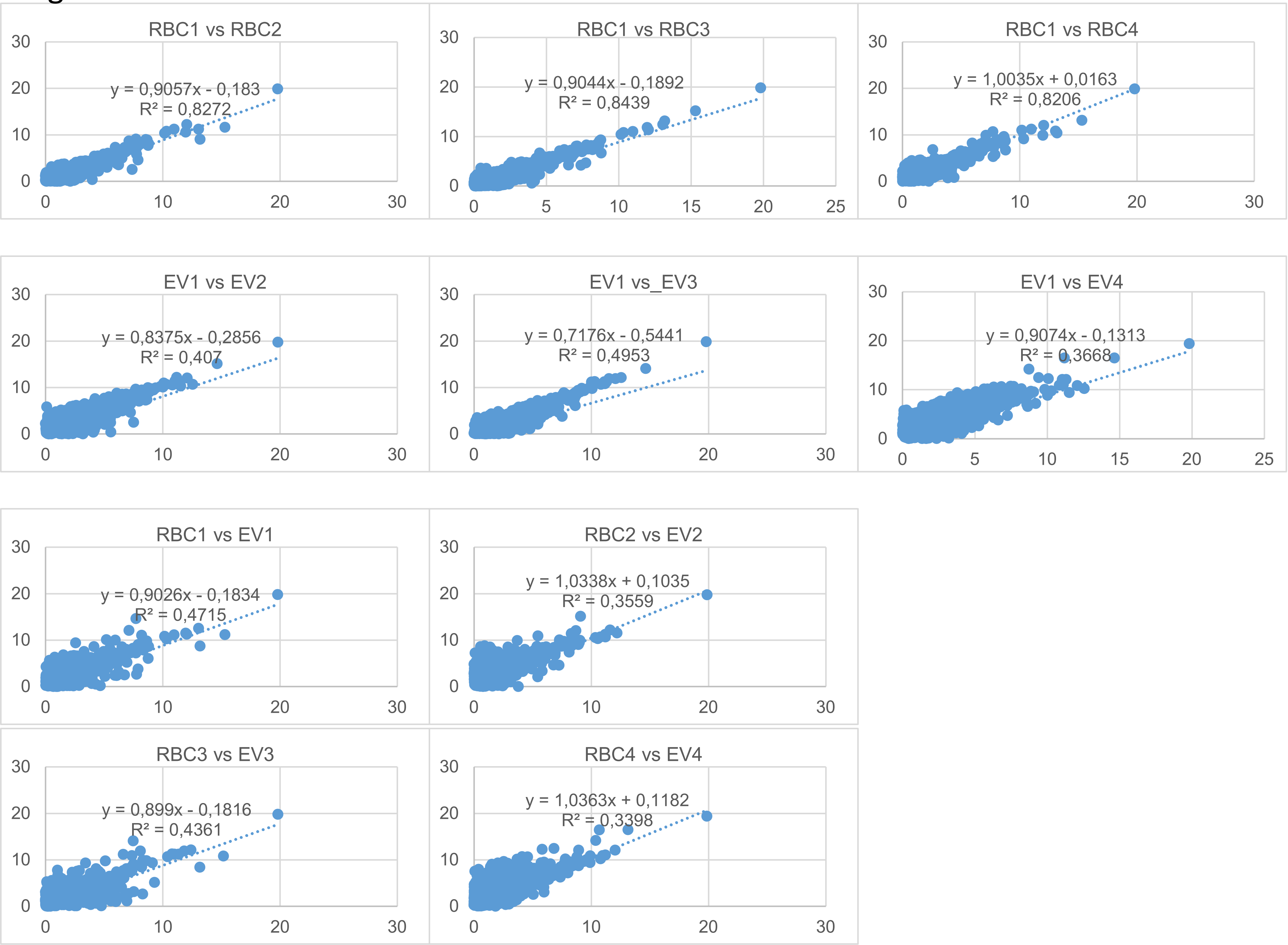
RBC1 vs RBC2
y = 0,9057x - 0,183
R² = 0,8272
RBC1 vs RBC3
y = 0,9044x - 0,1892
R² = 0,8439
RBC1 vs RBC4
y = 1,0035x + 0,0163
R² = 0,8206
EV1 vs EV2
y = 0,8375x - 0,2856
R² = 0,407
EV1 vs_EV3
y = 0,7176x - 0,5441
R² = 0,4953
EV1 vs EV4
y = 0,9074x - 0,1313
R² = 0,3668
RBC1 vs EV1
y = 0,9026x - 0,1834
R² = 0,4715
RBC2 vs EV2
y = 1,0338x + 0,1035
R² = 0,3559
RBC3 vs EV3
y = 0,899x - 0,1816
R² = 0,4361
RBC4 vs EV4
y = 1,0363x + 0,1182
R² = 0,3398

Figure S3.

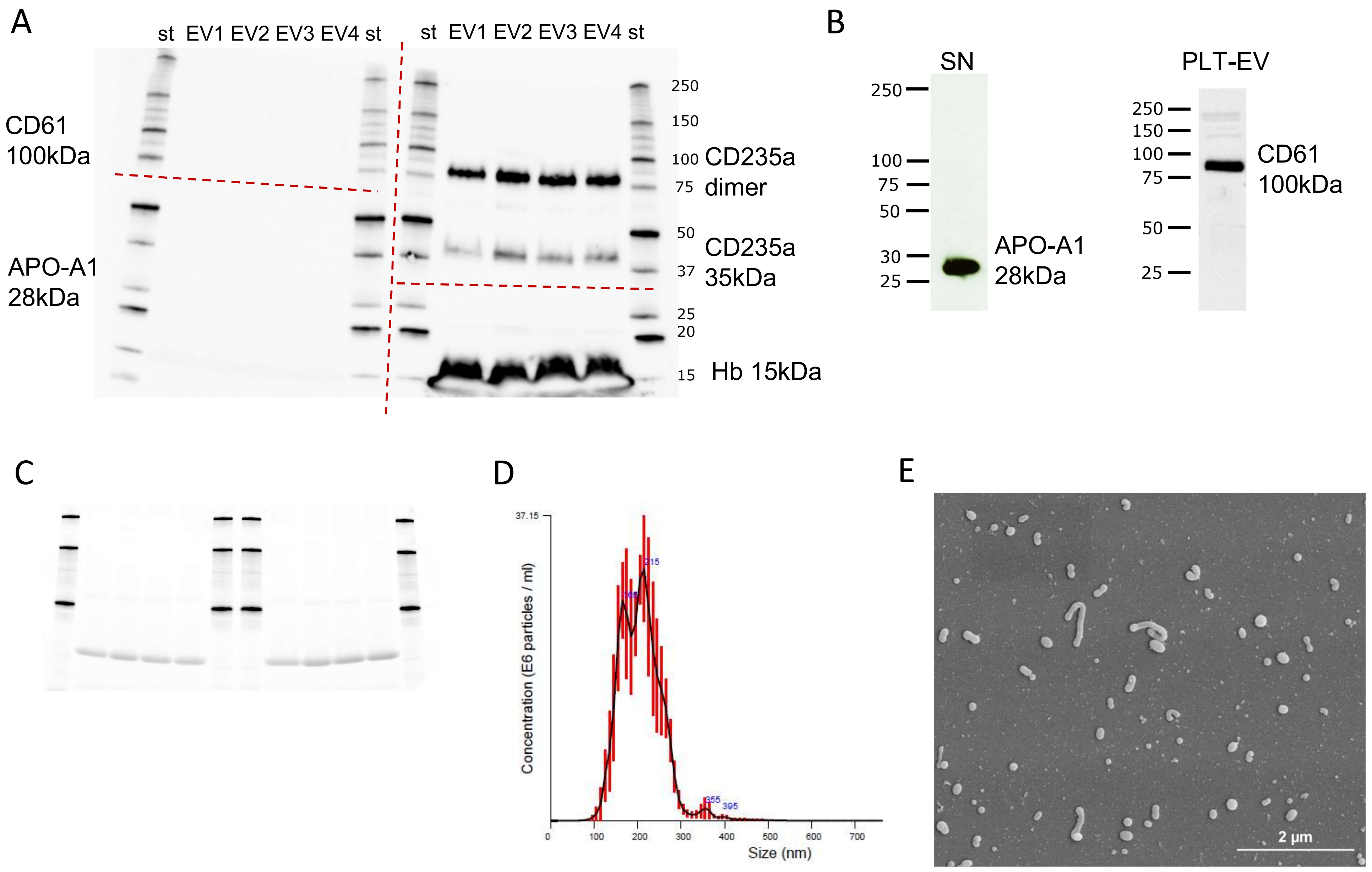
A
st EV1 EV2 EV3 EV4 st
st EV1 EV2 EV3 EV4 st
CD61
100kDa
APO-A1
28kDa
250
150
100
75
50
37
25
20
15
CD235a
dimer
CD235a
35kDa
Hb 15kDa
B
SN
250
100
75
50
30
25
APO-A1
28kDa
PLT-EV
250
150
100
75
50
25
CD61
100kDa
C
D
37.15
Concentration (E6 particles / ml)
215
355
395
0
100
200
300
400
500
600
700
Size (nm)
E
2 µm

Figure S4.

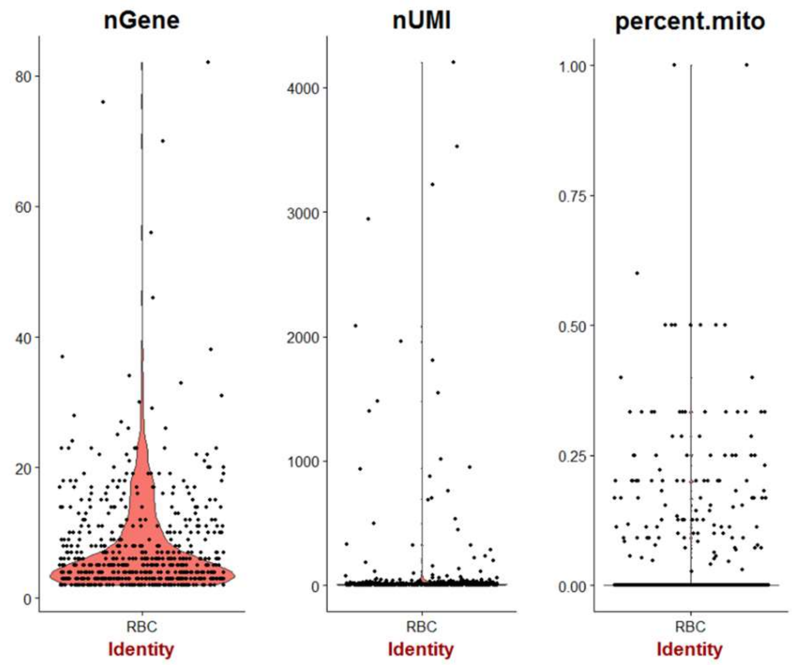
nGene
nUMI
percent.mito
80
60
40
20
0
4000
3000
2000
1000
0
1.00
0.75
0.50
0.25
0.00
RBC
Identity

Figure S5.

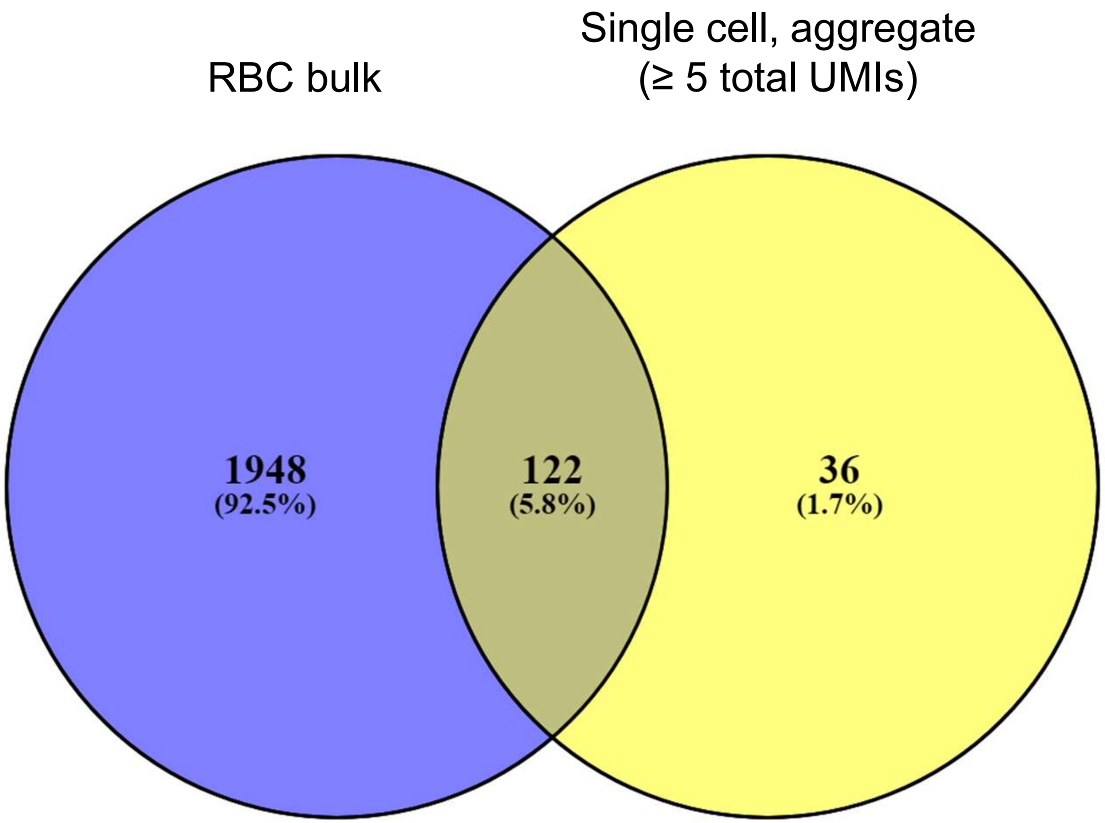

RBC bulk
Single cell, aggregate
(≥ 5 total UMIs)
1948
(92.5%)
122
(5.8%)
36
(1.7%)
